# The food environment in favelas is associated with the presence of arterial hypertension and diabetes in socially vulnerable women

**DOI:** 10.1017/S1368980025000175

**Published:** 2025-02-03

**Authors:** Luiz Gonzaga Ribeiro Silva-Neto, Risia Cristina Egito de Menezes, Juliana Souza Oliveira, Nathalia Paula de Souza, Thays Lane Ferreira dos Santos, Telma Maria de Menezes Toledo Florêncio

**Affiliations:** 1 Programa de Pós-Graduação em Nutrição, Escola Paulista de Medicina, Universidade Federal de São Paulo. R. Botucatu, 740. Vila Clementino, São Paulo, SP, 04023-062, Brasil; 2 Programa de Pós-Graduação em Nutrição, Faculdade de Nutrição, Universidade Federal de Alagoas. Avenida Lourival Melo Mota, s/n. Tabuleiro dos Martins, Maceió, AL, CEP: 57072-900, Brasil; 3 Curso de Nutrição, Centro Acadêmico de Vitória, Universidade Federal de Pernambuco, Rua Alto do Reservatório, s/n. Alto José Leal, Vitória de Santo Antão - PE, CEP: 55608-680, Brasil

**Keywords:** Poverty, Favela, Chronic diseases, Hypertension, Diabetes

## Abstract

**Objective:**

To evaluate the relationship between the food environment in favelas and the presence of arterial hypertension and diabetes among women in the context of social vulnerability.

**Design:**

A cross-sectional and partially ecological population-based study was conducted in a Brazilian capital city. The healthiness and availability of ultra-processed foods in the food environment were assessed through retailer audits using the AUDITNOVA instrument. The presence of diabetes and arterial hypertension was evaluated based on self-reported prior medical diagnosis. Logistic regression models were applied using generalised estimating equations, adjusted for age, education, race/skin colour and poverty status.

**Participants:**

1882 adult women of reproductive age (20–44 years).

**Results:**

It was found that 10·9 % of women were hypertensive and 3·2 % had diabetes. The likelihood of having diabetes and arterial hypertension decreases with higher levels of healthiness in the food environment (diabetes (OR: 0·25; 95 % CI: 0·07, 0·97)/arterial hypertension (OR: 0·45; 95 % CI: 0·24, 0·81)) and increases with greater availability of ultra-processed foods in their living area (diabetes (OR: 2·18; 95 % CI: 1·13, 4·21)/arterial hypertension (OR: 1·64; 95 % CI: 1·09, 2·47)).

**Conclusions:**

These results suggest that characteristics of the consumer food environment have a significant effect on the occurrence of chronic diseases among socially vulnerable women, adding to the existing evidence in the literature and highlighting the need for integrated health care.

Chronic non-communicable diseases (NCD), such as arterial hypertension and diabetes, represent one of the main causes of global morbidity and mortality, disproportionately affecting vulnerable populations in low- and middle-income countries^(1,2)^. Characterised by a gradual onset and prolonged evolution, these multifactorial conditions are associated with socio-economic factors, inadequate dietary patterns and limited access to health services^(2,3)^.

In Brazil, social inequalities amplify the effects of NCD, especially in contexts of urban poverty, such as favelas^(1,4,5)^. These areas have a high population density and are home to around 16 million Brazilians, with the highest proportion of women in the Northeast region of the country^(6)^. In addition, they suffer from social and health vulnerability, high rates of food insecurity and limited access to healthy food or its low quality^(2,4,6,7)^.

Although the national literature points to growing trends in ultra-processed foods (UPF) consumption in the general population, there is a lack of evidence specifically linking the food environment of areas with marked inequalities, such as favelas, to NCD^(8,9)^. However, the combination of low income, an obesogenic food environment, and increased exposure to UPF is associated with worse health outcomes in these populations^(10–12)^.

The focus on women in these communities is particularly relevant, as in addition to being more vulnerable to NCD^(5,12)^, they play key roles as caregivers and those primarily responsible for choosing and preparing food in the home^(13)^. The expansion of UPF consumption, which is widely available in these environments, plays a central role in this scenario^(9–11)^.

These foods, associated with aggressive marketing strategies and relatively greater affordability, have been linked to a higher risk of NCD^(7,8,10,11,14)^. Despite advances in food environment research in Brazil, few studies have explored how socio-economic conditions, combined with changes in global food systems, influence the dietary patterns and health outcomes of women in Brazilian favelas. These favelas are marked by a double burden of malnutrition, with simultaneous prevalence of food insecurity and obesity^(4,15)^.

In view of this, this study aims to assess the relationship between the food environment in favelas and the presence of arterial hypertension and diabetes among women in situations of social vulnerability. By filling gaps in knowledge about these health determinants, it is hoped that it will contribute to the formulation of interventions that address social inequalities and health patterns in these specific contexts.

## Methods

### Design and study location

Cross-sectional (individual data) and partially ecological (environmental component), population-based study conducted between October 2020 and May 2021 in favelas and urban communities in the city of Maceió, capital of the state of Alagoas, Northeast Brazil.

The Brazilian Institute of Geography and Statistics^(16)^ characterises favelas and urban communities as areas predominantly characterised by households with varying degrees of legal insecurity of tenure and at least one of the following criteria: absence or incomplete provision of public services; predominance of buildings, urban landscapes and infrastructure generally self-produced or guided by urban and construction parameters different from those defined by public authorities and location in areas with occupancy restrictions defined by environmental or urban legislation.

### Sample size and selection

Taking into account the estimated 24 614 adult women of reproductive age (20–44 years) in the ninety-four favelas and urban communities of Maceió, along with a prevalence of 26·3 % for arterial hypertension among women aged 18 years and older in Maceió^(17)^, adopting a margin of error of 2 % and a CI of 95 %, it would be necessary to recruit at least 1731 women. The sample size calculation was performed using the StatCalc v. 7.2.5.0 program (Center for Disease Control, Atlanta, EUA).

**Figure 1.**
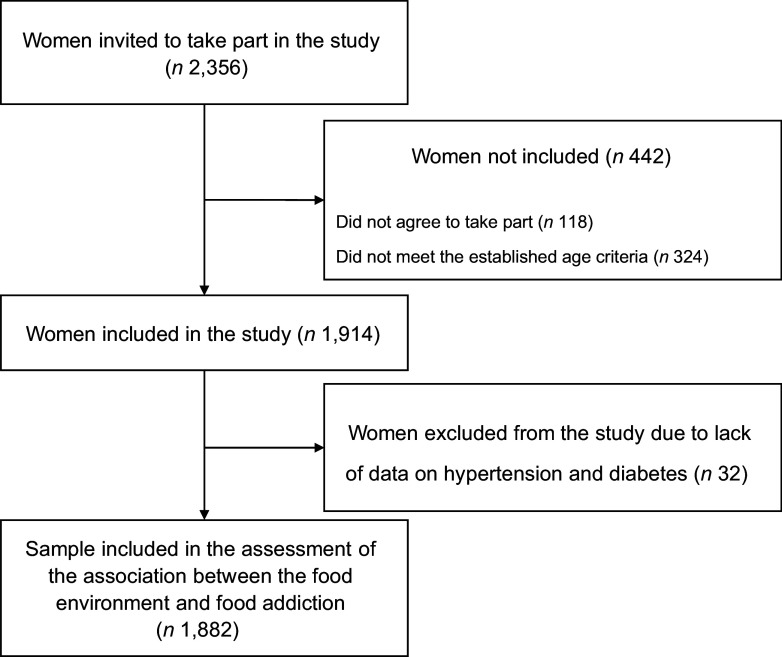
Flow chart for the inclusion of study participants.

**Figure 2.**
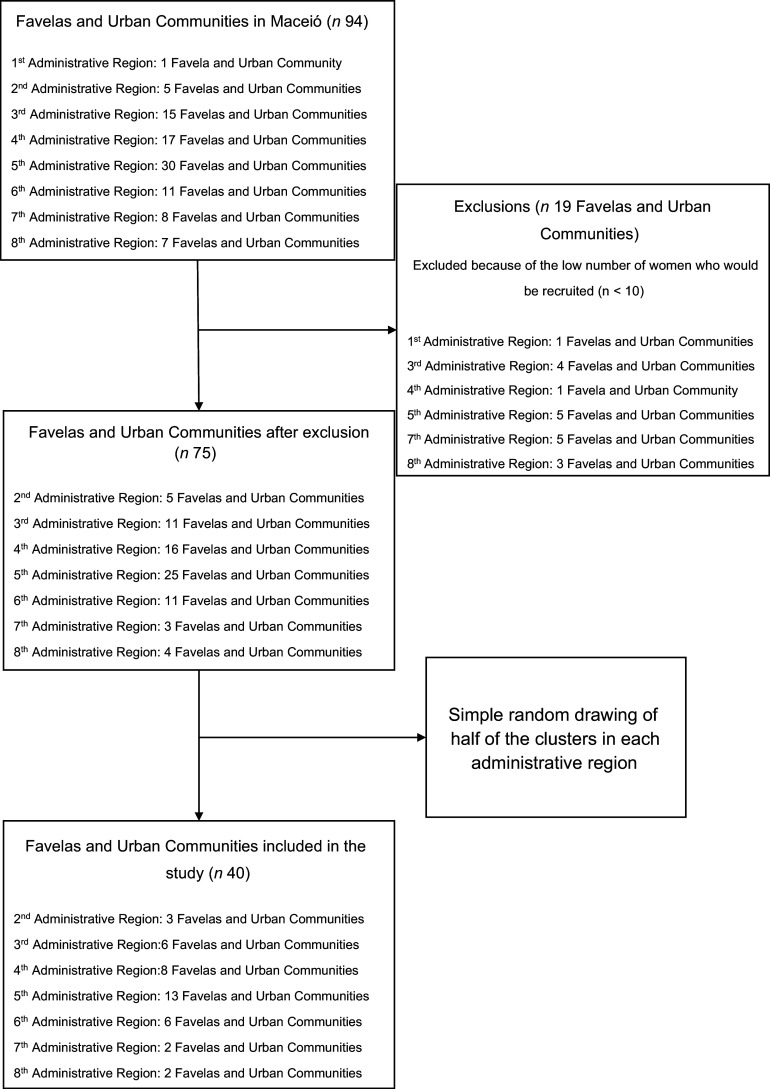
Flow chart for selecting the favelas and urban communities included in the study.

A total of 2356 women were invited to participate in the study, of which 1882 were included. The inclusion process is described in Figure 1. The women were recruited from forty favelas and urban communities, randomly chosen according to the criteria presented in Figure 2.

The study used a three-stage probabilistic cluster sampling design: (i) Favelas and urban communities were selected randomly and proportionally from each of the seven administrative regions of Maceió studied. (ii) Census tracts: One was randomly selected from each favela and urban community. (iii) Streets: One street was randomly selected for data collection in each census tract evaluated.

All households on the selected street were visited, and when necessary, neighbouring ones were included until the corresponding sample size for the area was completed. All households where at least one adult woman of reproductive age (20–44 years) resided were included, with data collected from one woman per household. Pregnant women and those with any disability that compromised their food intake or prevented them from taking part in the interview or understanding the survey questionnaires were not included.

### Data collection

#### Socio-demographic and health variables

To characterise the population, the following variables were collected: age (years), schooling (years of study) and race/skin colour (white, black, brown, yellow and indigenous). Monthly per capita family income was also assessed and classified according to the cut-off points for poverty (poverty – US$ < 91·90; and out-of-poverty US$ ≥ 91·90). Values converted from reais to US dollars, considering the average dollar exchange rate between October 2020 and May 2021 – R$5·43)^(18)^.

#### Dependent variables - arterial hypertension and diabetes

The presence of arterial hypertension and diabetes was assessed through the women’s self-report of a previous medical diagnosis of these chronic conditions. To determine the presence of arterial hypertension, the following questions were asked: ‘Has any doctor ever told you that you have high blood pressure?’ and ‘Has any doctor asked you to take any medication to lower your high blood pressure?’ The second question was only asked of women who answered ‘yes’ to the first.

To determine the presence of diabetes, the following questions were asked: ‘Has a doctor ever told you that you have diabetes?’ and ‘Has a doctor ever asked you to take any medication to control diabetes?’. The second question was only asked of women who answered ‘yes’ to the first.

Those who answered ‘yes’ to the two questions relating to each disease were considered to have a diagnosis of arterial hypertension and diabetes. This method aligns with practices in other research studies^(17,19)^.

#### Independent variable - consumer’s food environment

All formal and informal retail businesses within a 400-m buffer were audited, a distance deemed suitable for assessing the relationship between the food environment and health outcomes^(20)^. This buffer was calculated from the midpoint of the streets selected for data collection in the favela under study. A total of 624 food retail establishments were audited.

The audit was conducted using the AUDITNOVA instrument, validated for food retail businesses in Brazil, which evaluates factors such as availability, price, variety and advertising strategies in food retail^(21)^. As recommended by Borges et al.^(21)^, to characterise the food environment, the primarily marketed food group in each establishment was determined. This involved counting the number of shelves, displays and counters for each food group category (Fresh/Minimally Processed Foods; Culinary Ingredients; Processed Foods; UPF). The food group with the largest display area was considered the primary marketed group in the establishment.

From the data collected in the audit process, it was possible to calculate the healthiness score of the consumer’s food environment, composed of two dimensions: (1) food dimension (score from –27 to 56), formed by the indicators of availability and promotional price of all audited foods and beverages and (2) environmental dimension (score from –18 to 15), composed of the indicators advertising/information and placement of advertisements within the stores^(22)^.

Finally, the values obtained in the food and environmental dimensions were summed to determine the healthfulness score, which ranges from –46 to 71 points. These scores were standardised on a scale from 0 to 100, where higher scores indicate healthier establishments. The average final score for each favela and urban community was also stratified into tertiles: the first tertile indicates low healthfulness, the second tertile indicates intermediate healthfulness and the third tertile indicates high healthfulness.

The availability of UPF for each of the audited businesses was also calculated, following the proposal of Serafim et al.^(23)^. For this procedure, all eighteen UPF available in AUDITNOVA were considered, and they were grouped into five subcategories: (i) sausages – sausage and pork sausage; (ii) bakery products, biscuits and snacks – bread, breakfast cereals, snacks and cookies; (iii) sweets – ice cream, chocolates and candies; (iv) sugary drinks –canned soda, 2L soda, zero/light/diet soda, nectar, mix of soda and milk drink and (v) ready-to-eat foods – ready-to-eat pizza, seasoning mix and instant noodles.

The scoring of the subcategories was constructed based on the number of available foods in each audited business: processed meats – 2 items (score 0–11); bakery products, biscuits and snacks – 4 items (score 0–22); sweets – 3 items (score 0–17); sugary beverages – 6 items (score 0–33) and ready-to-eat foods – 3 items (score 0–17). Scores were standardised on a scale from 0 to 100 points, where a higher number of UPF available in each subcategory corresponded to a higher score, as utilised in the study by Serafim et al.^(23)^. Consequently, the average final score for each favela was determined, which was further stratified into tertiles: the first tertile indicating low availability, the second intermediate and the third high availability.

#### Spatial data

The geographic coordinates (latitude and longitude) of all audited retail businesses were collected using the Google Earth v. 9.3.25.5 application (Google, United States), positioned 1 meter from their main entrance. Subsequently, this data were entered into the QGIS 3.16.15 software (Open Source Geospatial Foundation, Chicago, United States).

After this procedure, the layer containing the previously calculated buffer was overlaid with another layer containing establishment-level data to calculate average values of measures assessing the healthiness and availability of UPF. This provided information for each favela and urban community.

### Data analysis

Descriptive analyses were conducted for both individual characteristics and the food environment, with continuous variables presented as mean and sd and categorical variables as absolute and relative frequencies. Variables describing the food environment were analysed continuously and presented median and interquartile ranges.

The presence of arterial hypertension and diabetes was considered as dependent variables. Independent variables included characteristics of the food environment related to healthiness (categorised values into tertiles: low healthiness, intermediate healthiness and high healthiness) and the availability of UPF (categorised values into tertiles: low availability, intermediate availability and high availability).

The association analysis was conducted using binary logistic regression through generalised estimating equations. The association was estimated by OR and their respective 95 % CI. For this procedure, three evaluation models were created: Model 1 included the healthiness of the environment, Model 2 included the availability of UPF in the environment and Model 3 included both the healthiness and availability of UPF in the environment. The models were adjusted for the following confounding variables: age (years), years of schooling, race/skin colour and poverty status. Analyses were performed using the statistical software Jamovi Computer Software (Version 2.3.28, The jamovi project, Sydney, Australia). A significance level of <5 % was adopted.

## Results

The socio-demographic and health characteristics of women included in this study are available in Table 1. Regarding arterial hypertension and diabetes, 10·9 % and 3·2 % of women had these conditions, respectively. The mean age and years of formal education were found to be 31·0 years and 8·1 years, respectively. We observed that 61·1 % of the population self-identified as mixed-race (brown), and 75·8 % were living in poverty.

**Table 1.** Socio-demographic and health characteristics of women living in slums and urban communities in Maceió, northeast Brazil, 2020/2021 (*n* 1882)

Variables	*n*	%
Diagnosis of arterial hypertension		
Yes	206	10·9
No	1676	89·1
Diagnosis of diabetes		
Yes	60	3·2
No	1822	96·8
Age (years)		
Mean	31·0	
sd	7·9	
Years of schooling		
Mean	8·1	
sd	3·7	
Race/skin colour		
White	255	13·5
Black	298	15·8
Brown	1149	61·1
Yellow	148	7·9
Indigenous	17	0·9
Not informed	15	0·8
Poverty situation*		
Yes	1427	75·8
No	455	24·2

* Assessed by monthly household income per capita (poverty – US$< 91·90; and out of poverty US$ ≥ 91·90. Values converted from reais to US dollars, considering the average dollar exchange rate between October 2020 and May 2021 – R$5·43)^(18)^.

Regarding the food environment, it was identified that 31·4 % and 2·9 % of the evaluated commercial establishments primarily sold fresh/minimally processed foods and processed foods, respectively, while 65·7 % primarily sold UPF. The median healthiness score of the food environment was 43·9 (IQR 42·3–46·3) points, both in the presence and absence of arterial hypertension. Similarly, when assessing diabetes, the highest median score was observed in the absence of this NCD, 43·9 (IQR 42·3–46·3) points (Table 2). For the evaluation of UPF availability, the highest median score found was 41·0 (IQR 36·6–48·5) points, both for the presence of arterial hypertension and diabetes (Table 2).

**Table 2.** Characteristics of the consumer’s food environment, according to the presence of arterial hypertension and diabetes in socially vulnerable women from socially vulnerable regions of Maceió, northeast Brazil, 2020/2021 (*n* 624)

			Arterial hypertension				Diabetes			
	Food environment		Yes		No		Yes		No	
Variables	Median	IQR	Median	IQR	Median	IQR	Median	IQR	Median	IQR
Healthiness score*	43·9	42·3–46·3	43·9	42·3–46·3	43·9	42·3–46·3	42·9	42·3–45·7	43·9	42·3–46·3
Food component*	40·9	40·9–43·0	40·9	37·8–43·0	40·9	37·8–43·0	39·0	38·1–42·7	40·9	37·8–43·0
Environmental component^*^	47·8	46·6–48·7	48·5	46·6–49·1	47·8	46·6–48·7	47·6	46·3–48·5	47·8	46·6–48·7
UPF availability score^†^	41·0	36·6–48·5	41·0	36·6–48·5	40·3	36·7–48·5	41·0	36·6–48·5	40·2	36·6–48·5

IQR, interquartile range; UPF, ultra-processed foods.

* Assessed according to the proposal by Borges; Gabe; Jaime^(22)^.

† Assessed according to the proposal by Serafim et al.^(23)^.

Table 3 describes the association between the food environment and the presence of arterial hypertension. Through the adjusted analyses, the healthiness classified as intermediate and high of the food environment decreased the chances of women having arterial hypertension by up to 55 % (OR: 0·45; 95 % CI: 0·24, 0·81) and 62 % (OR: 0·38; 95 % CI: 0·17, 0·83), respectively (Model 1). It was also possible to observe that the availability of intermediate (OR: 1·57; 95 % CI: 1·07, 2·32) and high (OR: 1·64; 95 % CI: 1·09, 2·47) UPF in the food environment increased the odds of women having arterial hypertension by approximately 1·6 times (Model 2). When including both healthiness and availability measures of UPF in the food environment in the same model, it was identified that high healthiness decreases the odds of arterial hypertension by up to 63 % (OR: 0·37; 95 % CI: 0·16, 0·86). Meanwhile, intermediate availability (OR: 1·74; 95 % CI: 1·08, 2·85) and high availability (OR: 1·75; 95 % CI: 1·13, 2·70) of UPF increased the odds of arterial hypertension by more than 1·7 times (Model 3).

**Table 3.** Associations between characteristics of the food environment and the presence of arterial hypertension in women living in slums and urban communities in Maceió, northeast Brazil, 2020–2021 (*n* 1882)

	Model 1			Model 2			Model 3		
Variables	OR	95 %CI	*P*-value	OR	95 %CI	*P*-value	OR	95 %CI	*P*-value
Healthiness score*									
T1	1·00						1·00		
T2	0·45	0·24, 0·81	0·008				0·84	0·47, 1·51	0·563
T3	0·38	0·17, 0·83	0·016				0·37	0·16, 0·86	0·021
Availability of AUP^†^									
T1				1·00			1·00		
T2				1·57	1·07, 2·32	0·022	1·74	1·08, 2·85	0·024
T3				1·64	1·09, 2·47	0·018	1·75	1·13, 2·70	0·011

UPF, ultra-processed foods.

The models were adjusted for age (years), years of schooling, race/skin colour and poverty status.

* Assessed according to the proposal by Borges; Gabe; Jaime^(22)^. T1: low healthiness; T2: intermediate healthiness; T3: high healthiness of the consumer’s food environment.

† Assessed according to the proposal by Serafim et al.^(23)^. T1: low availability; T2: intermediate availability; T3: high availability of UPF in the consumer’s food environment.

In Table 4, it was also identified that high healthiness in the food environment decreased the odds of women having diabetes by up to 75 % (OR: 0·25; 95 % CI: 0·07, 0·97) (Model 1), while high availability of UPF in the food environment increased the odds by up to 2·2 times (OR: 2·18; 95 % CI: 1·13, 4·21) (Model 2). When both healthiness and availability measures of UPF in the food environment were included in the same model, it was observed that high healthiness reduced the odds of diabetes by up to 60 %, while intermediate and high availability of UPF increased the odds by up to 2·8 times (OR: 2·78; 95 % CI: 1·20, 6·45) and 2·2 times (OR: 2·22; 95 % CI: 1·04, 4·77), respectively (Model 3).

**Table 4.** Associations between characteristics of the food environment and the presence of diabetes in women living in slums and urban communities in Maceió, northeast Brazil, 2020–2021 (*n* 1882)

	Model 1			Model 2			Model 3		
Variables	OR	95 %CI	*P*-value	Variables	OR	95 %CI	*P*-value	Variables	OR
Healthiness score*									
T1	1·00						1·00		
T2	0·41	0·15, 1·09	0·074				0·57	0·25, 1·31	0·187
T3	0·25	0·07, 0·97	0·045				0·40	0·17, 0·97	0·042
Availability of AUP^†^									
T1				1·00			1·00		
T2				1·76	0·94, 3·28	0·076	2·78	1·20, 6·45	0·017
T3				2·18	1·13, 4·21	0·021	2·22	1·04, 4·77	0·040

UPF, ultra-processed foods.

The models were adjusted for age (years), years of schooling, race/skin colour, and poverty status.

* Assessed according to the proposal by Borges; Gabe; Jaime^(22)^. T1: low healthiness; T2: intermediate healthiness; T3: high healthiness of the consumer’s food environment.

† Assessed according to the proposal by Serafim et al.^(23)^. T1: low availability; T2: intermediate availability; T3: high availability of UPF in the consumer’s food environment.

## Discussion

The findings presented in this study highlight the relationship between the food environment and arterial hypertension and diabetes among socioeconomically vulnerable populations. This relationship can potentially be replicated in other areas of Brazil, Latin America and other low- and middle-income countries around the world. An inverse association was identified between the environmental health index and arterial hypertension and diabetes, while the high availability of UPF was positively associated with these conditions in vulnerable women.

The prevalence of arterial hypertension (10·1 %) and diabetes (3·2 %) in the study is lower than the national (arterial hypertension, 29·3 %; diabetes, 11·1 %)^(17)^ and global (arterial hypertension, 19·1 %; diabetes, 23·8 %) averages^(24)^, indicating the importance of considering this vulnerable context in the future perspective of health investments^(25)^. From this increase, especially in more vulnerable areas, it is possible to detect NCD, such as arterial hypertension and diabetes, at an early stage^(26)^. However, there are significant barriers to the prevention and adequate treatment of NCD in Brazil, including low health coverage, an insufficient number of health professionals, and the need for more priority to promote an adequate and healthy diet^(27,28)^.

Therefore, actions related to chronic diseases must take into account the influence that the food environment has on food choices and health outcomes, which are affected by factors such as availability, variety, price, quality, advertising and marketing strategies and household access to food^(21,23)^, as well as socioeconomic, cultural, territorial, biological and individual conditions^(4,29)^.

The food environment in economically vulnerable areas lacks commercial establishments that offer healthy, quality food options, as identified in this study, in which the majority of the establishments evaluated sold mainly UPF^(4,15)^. As a result, economically vulnerable populations increasingly have access to cheaper food of lower nutritional quality, which leads to the adoption of inadequate eating habits^(30)^ and is related to the current epidemiological profile of the population^(29)^.

Our results show that the greater availability of UPF in the food environment is positively associated with the presence of arterial hypertension and diabetes. Worryingly, there is widespread marketing, distribution and consumption of UPF with high sugar, Na and saturated fat content, high-calorie concentration, high glycaemic index and low fibre content in Brasik^(8,9)^.

These associations highlight the need for interventions that promote healthy food choices and reduce the presence of foods known to be harmful to health, such as UPF^(31)^. Therefore, at the heart of addressing the food environment in the context of NCD is the urgent need to transform the dominant food system and, consequently, the food environment. This transformation involves strengthening local food production and increasing the availability of and access to healthy foods that are part of regional food cultures, thus helping to reduce dependence on UPF and protect the health of the population^(32,33)^.

Our results corroborate the observations of studies that have evaluated the influence of the food environment on eating behaviour, directly impacting the occurrence of NCD^(34)^, especially CVD^(29)^. In fact, the characteristics of the food environment can influence a population’s eating patterns in various ways^(4)^, particularly in the consumer’s food environment, where the availability, easy access and predominant presence of UPF can lead people to consume these foods more frequently, adopting unhealthy eating patterns^(30)^.

In addition, the characteristics of UPF affect the development and worsening of diseases in a continuous and chronic process since prolonged consumption of these foods leads to adaptations in eating behaviour that prevent individuals from stopping or reducing their intake^(35)^. Energy intake from these foods, especially among socially vulnerable women, is increasing^(8)^, which leads to an increase in body adiposity, especially visceral fat deposition, which tends to trigger pro-inflammatory processes through the release of substances such as adipokines, which raise blood pressure and negatively influence insulin action^(36)^.

Additional mechanisms related to UPF deserve attention. Chemical additives not used in traditional food preparation (emulsifiers, non-nutritive artificial sweeteners and thickeners) are added to these products, with already recognised negative cardiometabolic effects^(37)^. In addition, UPF are hyperpalatable, relatively cheap, practical and widely available, which favors high consumption in the general population^(38)^. In this context, broadening the focus to NCD and the environment in which people live makes it possible to identify the challenges to better address these conditions. Thus, the results of this study are useful for supporting the adoption of public policies that enable people to adopt adequate and sustainable dietary practices.

In contrast, our findings also highlight that a healthier food environment, with a greater presence of fresh and minimally processed foods, showed a negative association with the presence of arterial hypertension and diabetes. Being in a healthier food environment reflects positively on diet quality^(39)^, helping to prevent non-communicable chronic diseases^(40)^.

This is because physical and financial access to fresh food can encourage healthy food choices, positively influencing eating habits, especially among socioeconomically vulnerable populations^(32)^. We also emphasise that dietary recommendations for the prevention and control of NCD are difficult to adopt and maintain in an environment that promotes and encourages habits and attitudes contrary to these practices^(41)^.

In this context, public policies should aim to facilitate the adoption of healthy and sustainable practices^(42)^, incorporating environmental and lifestyle factors, such as taxing UPF^(43)^, subsidies for the production of fresh/minimally processed foods^(33)^ and front-of-pack labeling, which helps consumers with clear information about the composition of foods^(44)^. In addition, it is essential to regulate marketing strategies used to promote UPF and implement permanent policies, such as school feeding programs, encouraging healthy habits from an early age. However, changing the food environment through regulatory measures faces challenges, such as a lack of political support, food industry strategies, financial limitations and the population’s resistance to accepting these changes^(31)^. In this sense, food education has emerged as a crucial tool for raising public awareness of the importance of gradually transforming the food environment, reducing the consumption of UPF and promoting healthier choices.

At the same time, it is necessary to expand initiatives that guarantee fair access to healthy food, considering the financial and structural barriers faced by vulnerable populations^(43)^. The FAO High-Level Panel of Experts on Food Security and Nutrition emphasises that inadequate food environments compromise food security, especially in vulnerable urban populations, contributing to the double burden of malnutrition and increased NCD^(45)^. Key recommendations include strengthening access to fresh and healthy food, encouraging local production and distribution, regulating marketing practices that promote UPF and developing integrated policies that address structural inequalities in food supply^(45)^.

Our study has strengths and limitations. Our strengths include the novel association found between consumer food environment characteristics and two NCD among women living in favelas in a Brazilian capital. In addition, our study assessed the food environment through audits in commercial establishments using an instrument validated for the Brazilian context, which is an effective method for assessing the quality of the food environment. This approach provided a systematic, standardised and comprehensive assessment^(46)^.

As limitations, we can point to the self-reported medical diagnoses of arterial hypertension and diabetes by the participants, especially considering their low level of education. For this reason, results can be underreported. Some women may have arterial hypertension or diabetes and have not yet been diagnosed. However, as mentioned above, this method is used by the Brazilian government to assess the prevalence of these two diseases in the country. This form of assessment has been used in Brazil for many years, with the entire population, regardless of their education or economic situation. In addition, our sample is made up of women living in poverty, which can limit access to healthcare and medical diagnosis of these conditions. These factors may have influenced the relatively low prevalence of diabetes in our sample.

However, a study evaluating individuals with a medical diagnosis of diabetes found that 75 % of them accurately reported their diagnosis^(47)^. Self-reported arterial hypertension, on the other hand, has high specificity (88 %) and moderate sensitivity (77 %) in Brazilian studies, demonstrating its validity as a population screening tool, especially in homogeneous contexts, such as vulnerable communities, where access to formal diagnoses is limited^(48)^. Studies also point to greater congruence in reports among women, possibly due to the central role they play in family health care, which reinforces the method’s reliability in female populations. Thus, self-reported arterial hypertension is a valid strategy for identifying health trends in groups with specific socioeconomic and cultural characteristics, such as women in slums^(48,49)^.

In addition, the associations found between food environment variables and the health conditions analysed should be interpreted with caution. Cross-sectional studies’ limitations include possible confounding factors and different time intervals between exposure and results. Therefore, although the study allows for the generation of new hypotheses and contributes to a comprehensive analysis in conjunction with other available scientific evidence, it reduces its ability to establish causality. We recommend conducting prospective studies to further explore the causality between the characteristics of the food environment and the health outcomes assessed.

In conclusion, our results suggest that the characteristics of the consumer’s food environment (lower healthiness index and high availability of UPF) significantly influence the prevalence of arterial hypertension and diabetes, especially among socially vulnerable women. Improving the food environment, with lower availability of UPF and greater access to healthy foods, is an intersectoral strategy that can contribute significantly to preventing and reducing the prevalence of arterial hypertension and diabetes in vulnerable women.
